# A comparison of seasonal rainfall forecasts over Central America using dynamic and hybrid approaches from Copernicus Climate Change Service seasonal forecasting system and the North American Multimodel Ensemble

**DOI:** 10.1002/joc.7969

**Published:** 2023-01-06

**Authors:** Katherine M. Kowal, Louise J. Slater, Alan García López, Anne F. Van Loon

**Affiliations:** ^1^ Department of Geography and the Environment University of Oxford Oxford UK; ^2^ Sección de Aplicaciones Climáticas en el Departamento de Investigación y Servicios Meteorológicos Instituto Nacional de Sismología, Vulcanología, Meteorología e Hidrología (INSIVUMEH) Guatemala City Guatemala; ^3^ Institute for Environmental Studies (IVM) Vrije Universiteit Amsterdam Amsterdam The Netherlands

**Keywords:** Central America, dynamic, ensemble, forecasting, hybrid, precipitation, predictability, seasonal

## Abstract

Seasonal rainfall forecasts provide information several months ahead to support decision making. These forecasts may use dynamic, statistical, or hybrid approaches, but their comparative value is not well understood over Central America. This study conducts a regional evaluation of seasonal rainfall forecasts focusing on two of the leading dynamic climate ensembles: the Copernicus Climate Change Service seasonal forecasting system (C3S) and the North American Multimodel Ensemble (NMME). We compare the multimodel ensemble mean and individual model predictions of seasonal rainfall over key wet season periods in Central America to better understand their relative forecast skill at the seasonal scale. Three types of rainfall forecasts are compared: direct dynamic rainfall predictions from the C3S and NMME ensembles, a statistical approach using the lagged observed sea surface temperature (SST), and an indirect hybrid approach, driving a statistical model with dynamic ensemble SST predictions. Results show that C3S and NMME exhibit similar regional variability with strong performance in the northern Pacific part of Central America and weaker skill primarily in eastern Nicaragua. In the northern Pacific part of the region, the models have high skill across the wet season. Indirect forecasts can outperform the direct rainfall forecasts in specific cases where the direct forecasts have lower predictive power (e.g., eastern Nicaragua during the early wet season). The indirect skill generally reflects the strength of SST associations with rainfall. The indirect forecasts based on Tropical North Atlantic SSTs are best in the early wet season and the indirect forecasts based on Niño3.4 SSTs are best in the late wet season when each SST zone has a stronger association with rainfall. Statistical predictions are competitive with the indirect and direct forecasts in multiple cases, especially in the late wet season, demonstrating how a variety of forecasting approaches can enhance seasonal forecasting.

## Graphical abstract

Dynamic, statistical, and hybrid approaches can be used to forecast seasonal rainfall. We compare approaches by evaluating how dynamic and hybrid forecasts from C3S and NMME perform relative to statistical predictions over Central America during key wet season months. Results reveal high forecast skill over the North Pacific and relatively low skill in eastern Nicaragua. The comparative value of hybrid and dynamic approaches over statistical methods is primarily in the early to middle wet season (May–August).

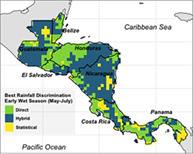

## 1 INTRODUCTION

Central America has been identified as a climate change hotspot at high risk of hydrometeorological extremes (Giorgi, 2006; Taylor *et al*., 2012; Almazroui *et al*., 2021). Drought and flooding events have devastated local communities in recent years (CEPAL, 2002; Marengo *et al*., 2014; Calvo‐Solano *et al*., 2018; Guevara‐Murua *et al*., 2018; Beveridge *et al*., 2019; Gotlieb *et al*., 2019). These events are unlikely to abate (Hidalgo *et al*., 2013; Hannah *et al*., 2017; Imbach *et al*., 2018; Almazroui *et al*., 2021), which makes extreme weather preparedness an important challenge going forward. Drought and flooding impacts are modulated by many factors, including social and economic conditions (e.g., Perez‐Briceno *et al*., 2016; Alfaro, Martínez *et al*., 2018). Early warnings based on forecasting do not provide a comprehensive solution to mitigate these impacts but can be one factor to reduce vulnerability when effectively communicated (Braman *et al*., 2013; de Perez *et al*., 2016; Kreibich *et al*., 2017; Golding *et al*., 2019; Goddard *et al*., 2020; Domeisen *et al*., 2022; White *et al*., 2022). Forecasts at the seasonal scale are useful to inform planning for the upcoming season, including decisions like crop choice and water supply and demand measures. Multiple efforts are underway to enhance seasonal forecast efficacy over Central America, providing stakeholders with rainfall information several months ahead. The Central American Climate Outlook Forum (CA‐COF), for instance, provides regional rainfall outlooks for national and sector‐specific planning (Donoso and Ramirez, 2001; Garcia‐Solera and Ramirez, 2012; Maldonado *et al*., 2013; Alfaro *et al*., 2016b). These outlooks are disseminated through several public forums, such as the Mesa Técnicas Agroclimáticas (MTAs) in Guatemala, which are local technical agroclimatic forums where farmers can interact with scientists to learn more about upcoming seasonal rainfall (INSIVUMEH, 2022b).

Regional evaluations of seasonal forecasts can help stakeholders choose which models are most effective to be used in these forums and identify opportunities to improve forecast skill. There are multiple ways to generate seasonal rainfall forecasts, including dynamic, statistical, and hybrid approaches (Amador and Alfaro, 2009; Hao *et al*., 2018). Dynamic forecasting approaches employ the predictions of physically‐based climate models (General Circulation Models [GCMs]) that are initialized at different states of the climate system (Bauer *et al*., 2015). These models are currently used by the CA‐COF, which often uses GCMs from the North American Multimodel Ensemble (NMME; Kirtman *et al*., 2014). Other dynamic forecasting systems are also publicly available, including the European ensemble from the Copernicus Climate Change Service (C3S; https://cds.climate.copernicus.eu/).

Statistical methods have also been used to estimate regional rainfall. Some studies for instance use Canonical Correlation Analysis (CCA) to relate observed SST in the Atlantic and Pacific oceans to regional rainfall, demonstrating the important role SST fields play in rainfall over Central America (Giannini *et al*., 2000; Alfaro, 2007; Maldonado *et al*., 2013; 2016; 2017; Alfaro *et al*., 2016a), including for predictions of low‐ and high‐rainfall events (e.g., rainfall above the 80th and below the 10th percentiles of the monthly climatology; Maldonado *et al*., 2013). Hybrid methods then combine dynamic and statistical methods (Slater *et al*., 2022). Some hybrid methods generate indirect rainfall forecasts by extracting related variables (e.g., SST) from the GCMs and then statistically translate those values to target variables (e.g., Alfaro *et al*., 2018; Strazzo *et al*., 2019; Colman *et al*., 2020). Indirect forecasts could perform well, as forecasts of large‐scale variability like SST over tropical domains are often a strength of the GCMs compared to their forecasts of more variable parameters like rainfall (Barnston *et al*., 2012; 2019; Saha *et al*., 2014). For example, Saha *et al*. (2014) found that one contributing model to NMME (CFSv2) had high anomaly correlations with SST in the Niño3.4 region (~0.82), while the average correlation with observed Northern Hemisphere precipitation rate over land is equal to 0.12. Using the GCMs for their strengths (e.g., SST estimates) and statistically translating those to a rainfall forecast could potentially improve their direct rainfall forecast skill.

In this paper we compare the following methods: dynamic methods that take rainfall forecasts directly from GCMs (direct), statistical methods based on observed SST values months ahead of a season (statistical), and hybrid methods that generate forecasts indirectly by using GCMs to predict SST over a target period and then statistically translate those values to a rainfall forecast in that same period (indirect). Evaluation of seasonal rainfall predictions over Central America is needed because GCMs that perform well globally may not necessarily forecast rainfall well over a given region (Hagedorn *et al*., 2005; Amador and Alfaro, 2009; Hidalgo and Alfaro, 2012; 2015; Doblas‐Reyes *et al*., 2013; Kharin *et al*., 2017; Almazroui *et al*., 2021). Some evaluations of GCMs have showcased their potential over Central America and in nearby regions (e.g., Kirtman *et al*., 2014; Weisheimer and Palmer, 2014; Carrão *et al*., 2018; Khajehei *et al*., 2018; Slater *et al*., 2019; Becker *et al*., 2020; Gubler *et al*., 2020; Kowal *et al*., 2021). Very few of these studies target Central America specifically and they typically compare one model, ensemble, or forecasting method, using different forecast verification metrics and time‐periods of evaluation. Furthermore, many of these studies baseline model skill against a climatological mean or a random forecast, which is standard practice for many skill metrics. It is relatively easy to outperform the climatological mean or a random forecast, so although multiple studies may show models outperform the climatological mean, for instance, it is difficult to choose between models and methods without one evaluation that compares them using the same time frame and verification metrics.

**FIGURE 1:**
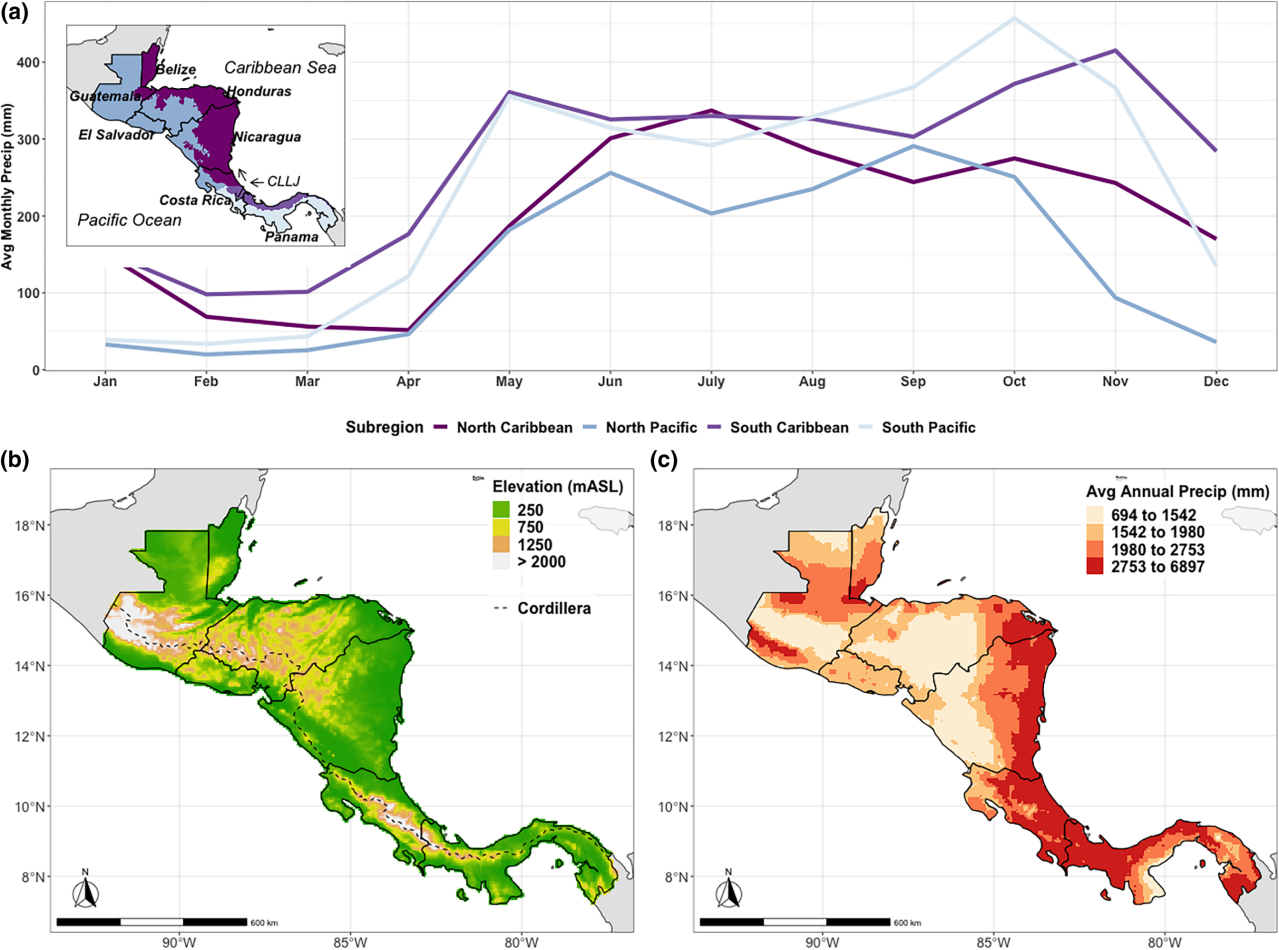
(a) Spatially averaged monthly rainfall between 1993 and 2016 (using CHIRPS) plotted by subregions, which are tagged in the inset map. The southern part of the isthmus is separated below 10°N when the CLLJ branches (Wang, 2007; Muñoz *et al*., 2008; Cook and Vizy, 2010; Hidalgo *et al*., 2015) and divided into a Caribbean and Pacific regime. The North Pacific includes the Pacific side of the Cordillera, the locations in Guatemala not located in the Caribbean climate regime as delineated by INSIVUMEH based on the Thornthwaite index (e.g., INSIVUMEH, 2022c), and the CADC, all of which experience a similar monthly rainfall distribution over the wet season. The CADC boundary is delineated here based on the climate risk index that includes relatively contiguous locations with a dry season that lasts over 4 months (IICACR, 2014). The North Caribbean then lies on the Caribbean side of the Cordillera excluding the additional locations demarcated as the North Pacific. (b) Elevation measured in meters above sea level (mASL) plotted with the Cordillera mountain range. (c) Average annual rainfall (CHIRPS) from 1993 to 2016 plotted spatially. [Colour figure can be viewed at wileyonlinelibrary.com]

Effective forecasts over Central America need to capture the region's seasonal rainfall cycle (Figure 1a). This cycle follows a bimodal distribution with peak rainfall primarily occurring in June and September typically separated by the mid‐summer dry period in July and August (Figure 1a; Magaña *et al*., 1999). This mid‐summer dry period arises due to a decrease in nearby SST, which limits deep convection activity and enables trade winds to intensify (Magaña *et al*., 1999). The seasonal distribution in rainfall also varies regionally with more extreme rainfall events often occurring on the Pacific slope during August–October compared to the Caribbean slope (Peterson *et al*., 2002; Taylor and Alfaro, 2005; Maldonado *et al*., 2018). The variability and intensity of seasonal rainfall is not uniform across the isthmus (e.g., Muñoz‐Jiménez *et al*., 2019) (see Figure 1a,c for plots of annual and monthly rainfall). For instance, the mid‐summer dry period is more intense in the Central American Dry Corridor (CADC—a regional drought hotspot; Gotlieb *et al*., 2019) compared to other locations (Figure 1a).

Central America is an important region for a regional forecast evaluation, as it can have both high forecast skill due to its location in the tropics, and low forecast skill because it is a relatively thin land bridge between two oceans and has relatively steep changes in terrain from the Cordillera (Figure 1b). One tropical teleconnection is the El Niño–Southern Oscillation (ENSO) in the eastern Pacific (Waylen *et al*., 1994; Spence *et al*., 2004; Amador *et al*., 2006; Poveda *et al*., 2006; Amador, 2008; Durán‐Quesada *et al*., 2017; Maldonado *et al*., 2018; Mariotti *et al*., 2020). ENSO is the dominant climate mechanism affecting rainfall over Central America through its modulation of moisture transport mechanisms like the Caribbean Low‐Level Jet (CLLJ) (Spence *et al*., 2004; Durán‐Quesada *et al*., 2017). Although ENSO is a well‐known indicator of rainfall over Central America, and its phases are publicized by meteorological bulletins to inform stakeholders (e.g., IMN, 2019), this phenomenon is unlikely to provide the full rainfall picture. El Niño phases of ENSO only explain some of the drought events in the CADC, for instance, and drought events differ in intensity between similar El Niño phases (Muñoz‐Jiménez *et al*., 2019; Kowal *et al*., 2021). The isthmus is affected by a complex interaction of weather patterns that arise from processes in both the Pacific and Atlantic oceans (Durán‐Quesada *et al*., 2020), including seasonal migration of the inter‐tropical convergence zone (ITCZ), tropical cyclones, and movement of the Atlantic warm pool (Enfield and Alfaro, 1999; Poveda and Mesa, 1999; Wang and Enfield, 2001; Amador *et al*., 2006; Wang, 2007; Amador, 2008; Hidalgo *et al*., 2015; Sori *et al*., 2015; Durán‐Quesada *et al*., 2017; 2020).

Evaluating whether the GCMs can improve on an ENSO‐based forecast is useful. ENSO is not only a known driver of regional rainfall but is also a known driver of skill of many GCMs (Yuan and Wood, 2013; Mo and Lyon, 2015; Scaife *et al*., 2019; Gubler *et al*., 2020), and it is worthwhile to explore how the skill of the direct and indirect forecasts differ from a statistical forecast based on ENSO alone. Some assessments indicate ENSO alone does not drive GCM skill (e.g., Gubler *et al*., 2020; Zhao *et al*., 2021). In their evaluation of SEAS5, the seasonal forecasting system produced by the European Centre for Medium‐Range Weather Forecasting (ECMWF), Gubler *et al*. (2020) used an ENSO‐based statistical forecast to test against SEAS5 performance over South America and demonstrated that SEAS5 skill differed from the ENSO‐based statistical forecast. Focusing on CFSv2, Zhao *et al*. (2021) also showed that the ENSO teleconnection contributed to some of the skill of CFSv2, especially in southern Central America in DJF, but that it was not a significant contributor to that model's skill in all parts of the isthmus, especially during other seasons when the ENSO teleconnection was less prominent. Both examples indicate that the GCMs can derive skill from other climatological mechanisms than ENSO, meaning they may outperform forecasts that only use this variable.

SST in the Atlantic is also important to regional rainfall (Enfield and Alfaro, 1999; Giannini *et al*., 2000; Waylen and Quesada, 2001; Taylor *et al*., 2002; Spence *et al*., 2004; Alfaro, 2007; Wang *et al*., 2008; Sori *et al*., 2015), as it modulates the magnitude of the mid‐summer dry period (Wang *et al*., 2008; Maldonado *et al*., 2016), among other influences over the region. Tropical North Atlantic anomalies (TNA; Enfield and Alfaro, 1999), for instance, are one of the inputs the CA‐COF uses to inform rainfall outlooks (Alfaro *et al*., 2016b). A systematic regional evaluation across multiple models and approaches compared to a statistical model that includes ENSO and TNA SSTs could inform how the skill of the GCM‐based forecasts (direct and indirect) differs from the performance of a statistical forecast that uses common SST indices known to influence regional rainfall.

This study explores how two of the leading dynamic forecasting ensembles, NMME and C3S, compare at seasonal rainfall prediction over Central America, considers how best to use their outputs, and investigates some of the climate mechanisms that may drive skill variability over the region. We do this by (a) evaluating how the skill of direct, indirect, and statistical forecasts varies spatially and temporally during key wet season periods, (b) comparing the relative value of using the C3S and NMME ensembles for direct or indirect forecasts to identify cases when they are significantly different from each other and a purely statistical forecast, and (c) exploring why some GCMs may perform better than others by investigating the relationship between the models' rainfall skill and the skill of their predictions of SST in some key climate regions.

## 2 DATA AND METHODS

### 2.1 Seasonal forecast scope

We focus on seasonal rainfall forecasts during key wet season months (May–October) across Central America, using 3‐month seasonal totals from the forecasting models initialized 1 month prior to the forecast, as is done operationally for rainfall outlooks produced by the CA‐COF (Donoso and Ramirez, 2001; Garcia‐Solera and Ramirez, 2012; Alfaro *et al*., 2016b). We choose three periods of interest that are used by the CA‐COF to assess different stages of the wet season: May–July (MJJ; early wet season), June–August (JJA; includes mid‐summer dry period), and August–October (ASO; late wet season). Seasonal forecasts are generated by taking the total rainfall over 3‐month periods. For instance, a forecast of total seasonal rainfall from MJJ is initialized in April, essentially adding rainfall from a 1‐month lead time for May, a 2‐month lead time for June, and a 3‐month lead time for July. Tercile deterministic forecasts, like the ones produced by the CA‐COF where predictions are made for below normal, normal, and above normal rainfall, are assessed using 33 and 66% thresholds in the seasonal rainfall distribution. Predictions of total seasonal rainfall above the upper 90% and below the lowest 10% thresholds are also assessed to better understand how predictive skill may change for rainfall extremes. We then assess the forecasts spatially and by subregion, comparing performance between the North Pacific, North Caribbean, South Pacific, and South Caribbean (as defined in Figure 1a).

### 2.2 Dynamic model selection and reference data

This evaluation focuses on two multi‐model ensembles: C3S and NMME. The European ensemble, C3S, has become publicly available as of 2018 and is hosted by the ECMWF at the Climate Data Store at daily and monthly timescales with 1–6 month lead times (https://climate.copernicus.eu/seasonal-forecasts). The leading North American ensemble, NMME (Kirtman *et al*., 2014), is hosted by the International Research Institute for Climate and Society at Columbia University (IRI) and is publicly available up to 11‐month lead times at monthly timescales (https://iridl.ldeo.columbia.edu/SOURCES/.Models/.NMME/). Several models contribute to both ensembles, of which 10 are selected for this evaluation (5 from NMME with 73 total members and 5 from C3S with 148 total members for hindcast analysis; see Table 1). The NMME models used in this study include multiple Phase II models that are operationally available and two newer updates since the 2019–2020 Phase II annual report—GFDL SPEAR and Cansips‐IC3 (Becker *et al*., 2020; NOAA, 2020). Five C3S models are selected based on publicly available spatial resolution (1°) and consistent initialization (1st of every month), as summarized in Table 1. Here, C3S and NMME are compared using the overlap between their publicly available data, which includes monthly temporal resolution and hindcasts from 1993 to 2016. For the purposes of this evaluation, when we refer to forecasts, we use forecasts as a general term for predictions but are using hindcasts and historical years.

**TABLE 1:** Summary of forecasting models in evaluation

NMME			
Contributing center	Model	Hindcast members	Reference
NCAR‐COLA/RSMAS	CCSM4	10	Gent *et al*. (2011)
ECCC‐CMC	Cansips‐IC3^a^	20	Lin *et al*. (2021) and Merryfield *et al*. (2013)
NASA‐GMAO	GEOSS2S, Version 2	4	Molod *et al*. (2020)
NOAA‐GFDL	SPEAR	15	Delworth *et al*. (2020)
NOAA‐NCEP	CFSv2	24	Saha *et al*. (2014)
Total models in analysis: 5; total members in analysis: 73			

C3S			
Contributing center	Model	Hindcast members	Reference
CMCC	System 35	40	Gualdi *et al*. (2020)
DWD	System 21	30	Fröhlich *et al*. (2021)
ECMWF	SEAS5	25	Johnson *et al*. (2018)
Meteo France	System 8	25	Batté *et al*. (2021)
UKMO	Glosea6	28	Davis *et al*. (2020)
Total models in analysis: 5; total members in analysis: 148			

*Note*: Contributing centre is listed next to model name, total number of members available for hindcasts, along with the reference for the model.

Abbreviations: NCAR‐COLA/RSMAS, U.S. National Center for Atmospheric Research Center for Ocean Land Atmosphere Studies/Rosenstiel School for Marine and Atmospheric Science at UMiami; ECCC, Environment and Climate Change Canada – Canadian Meteorological Centre; NASA‐GMAO, NASA Global Modelling and Assimilation Office; NOAA‐NCEP, NOAA National Center for Environmental Prediction; NOAA‐GFDL, NOAA Geophysical Fluid Dynamics Laboratory; CMCC, Fondazione Centro Euro‐Mediterraneo Sui Cambiamenti Climatici; DWD, Deutscher Wetterdienst; ECMWF, European Centre for Medium‐Range Weather Forecasts; UKMO UK Met Office.

^a^ Cansips‐IC3 is operationally treated as one but technically made up of the GEM5‐NEMO and CanCM4i‐IC3 models, each containing 10 members.

For the observed rainfall data, Climate Hazards Group InfraRed Precipitation with Station data (CHIRPS) total monthly rainfall data is used because it is a relatively common observational reference dataset in Central American studies (e.g., Alfaro *et al*., 2016a; Hidalgo *et al*., 2017), covers the entire time period of analysis (1993–2016), and is available at high spatial resolution ~0.05° (Funk *et al*., 2014). This dataset is compared against Multi‐Source Multi‐Weighted Ensemble Precipitation (MSWEP; Beck *et al*., 2017), Global Precipitation Climatology Center (GPCC; Schneider *et al*., 2018), but the difference in observational datasets is often smaller than the range of the forecasts (Figure S1, Supporting Information), and the forecast skill shows similar regional and seasonal variability across observational datasets, so we prefer CHIRPS (Figure S2). Optimum Interpolation Sea Surface Temperature data (OISSTv2—blended dataset that combines a variety of inputs, e.g., buoys, satellites; Reynolds *et al*., 2007) is used as the observed SST dataset to validate the ensemble SST hindcasts and generate the statistical predictions.

### 2.3 Forecast construction

#### 2.3.1 Direct forecasts from C3S and NMME using pyCPT

**FIGURE 2:**
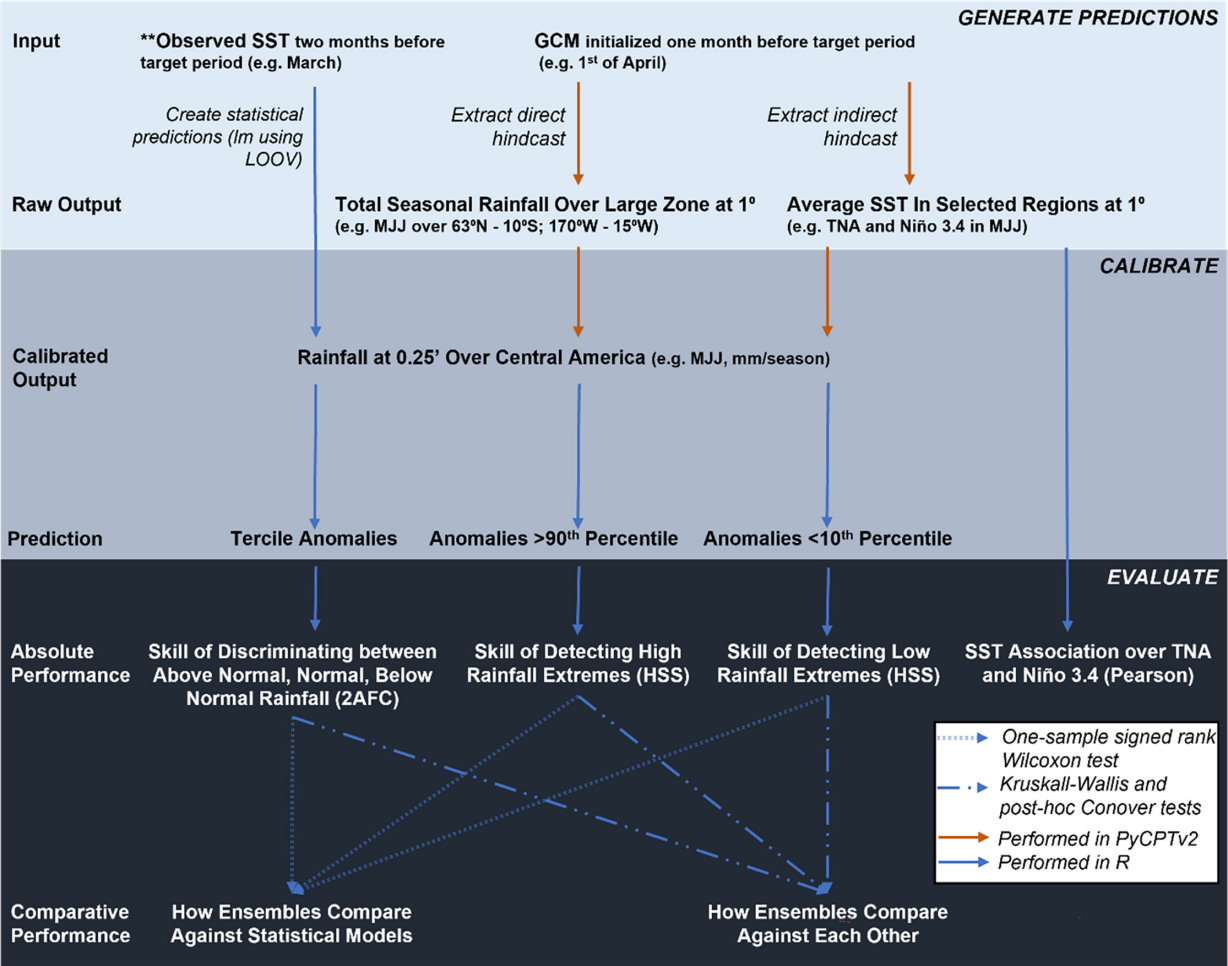
Diagram of methods employed to generate forecasts, calibrate them, and evaluate their performance. Statistical models: A linear model (lm) is generated using leave‐one‐out cross‐validation (LOOV) from 1981 to 2016 to train the models to make predictions over 1993–2016 based on 2‐month lagged SST for a specified region (e.g., Niño3.4 in March extracted to predict rainfall over May–July, MJJ). Direct models: Rainfall hindcasts are extracted at 1° from 1993 to 2016 over a larger region than Central America and then are bias‐corrected and calibrated using a canonical correlation analysis (CCA) model that translates the 1° raw hindcasts into 0.25° hindcasts over Central America. Indirect models: SST hindcasts are extracted at 1° from 1993 to 2016 over a target region (e.g., Niño3.4) and then are bias‐corrected and calibrated using a CCA model to then statistically translate the 1° raw SST hindcasts into 0.25° rainfall hindcasts over Central America. Once 0.25° raw predictions are made using each approach, the values are calculated as standardized anomalies and then percentiles of rainfall are extracted depending on the question (e.g., 0.33/0.66 for normal rainfall discrimination, 0.10 for low‐rainfall extremes, 0.90 for high‐rainfall extremes). Absolute skill is measured using Heidke skill score (HSS), 2 alternative forced choice (2AFC), and Pearson's correlation coefficient (Pearson). Comparative skill is tested by comparing the absolute skill between the models/forecasting approaches using Wilcoxon and Kruskal–Wallis/Conover statistical tests. [Colour figure can be viewed at wileyonlinelibrary.com]

To test the ensembles in an operational‐type setting, we evaluate the models and ensembles as they are produced with pyCPTv2 (Muñoz *et al*., 2019), a Python set of libraries that work as a wrapper for the Climate Predictability Tool developed by IRI (we used version 17.6.1; Mason *et al*., 2022), and are part of the NextGen methodology developed by IRI to improve climate services at subseasonal to seasonal timescales (Muñoz *et al*., 2019; WMO, 2020; Muñoz *et al*., 2020a). Here we provide a brief overview of the methodology, but other articles provide more details on the functionality (e.g., Muñoz *et al*., 2020a; Pons *et al*., 2021) and applications (e.g., Fernandes *et al*., 2020; 2022; WMO, 2020) of NextGen. Steps performed in pyCPT include (a) download raw 1° hindcasts of C3S and NMME GCMs from the IRI data library and (b) calibrate the hindcasts and transform them into 0.25° rainfall predictions over Central America using a Canonical Correlation Analysis (CCA) model (see Figure 2 for a flowchart illustrating how we generate, calibrate, and evaluate the predictions). The calibration procedure follows the pattern‐based CCA Model Output Statistics methodology described in Mason and Baddour (2008), which is designed to maximize the correlation of linear combinations between predictors and predictands, summarized as follows: (a) *correct biases* by applying integrated cosine latitude weighting to both predictor and predictands and transform them into normalized outputs; (b) *reduce dimensionality* of the lat/lon/time data sets into an *n* points by *n* years data set and handle missing datapoints (e.g., dropping data points with too many missing years of data and using *K*‐nearest neighbours to fill in missing data); (c) *separately fit a principle components analysis (PCA) time series* for predictors and predictands and calculate loadings by latitude and longitude to identify the number of empirical orthogonal function (EOF) modes for predictor/predictand; (d) *fit a CCA model* onto the two PCA time series—one for predictor and one for predictand; (e) *produce a prediction* by calculating the PCA scores for predictor values, apply the CCA prediction with those scores as inputs, and use the CCA loadings to reconstruct the rainfall prediction (for more details on CCA calibration in pyCPT, see Muñoz *et al*., 2020b; Martinez *et al*., 2022).

In practice, these steps are simplified using the pyCPT libraries, where the generation and calibration functions only require identification of the predictor field, the predictor variable (e.g., total seasonal rainfall or mean seasonal SST), the predictand variable (e.g., total seasonal rainfall), the maximum number of EOF modes for both predictor and predictand, and the maximum number of CCA modes to constrain how many comparisons the model runs (we choose maximum EOFx modes = 6, maximum EOFy modes = 5, and maximum CCA modes = 5). For the direct forecasts, we set the predictor field to precipitation with a larger grid box than Central America (63°N–10°S, 170°–15°W), which is selected based on the locations used for CCA in Maldonado *et al*. (2013) and includes both Pacific and Atlantic zones of variability. We export the calibrated models into R version 4.1.2 (R Core Team, 2021) to combine, evaluate, and visualize the results using several packages (Grolemund and Wickham, 2011; NCAR – Research Applications Laboratory, 2015; Wickham, 2016; Weigel, 2017; Pebesma, 2018; Pierce, 2019; Wickham *et al*., 2019, 2021; Huang and Zhao, 2020; Kassambara, 2020; Wickham and Seidel, 2020; Hijmans, 2021; Mangiafico, 2022; Pedersen, 2022; Pohlert, 2022). To create the ensembles, we use an unweighted combination of each calibrated model's mean to align with how the NextGen approach is currently deployed, and because the added benefit of a sophisticated post‐processing method like Bayesian Model Averaging is often limited where sample periods are under 30 years (Weigel *et al*., 2010; Delsole *et al*., 2013).

#### 2.3.2 Indirect and statistical forecasts in known teleconnection zones

We generate indirect and statistical forecasts using two known teleconnection regions—Niño3.4 (170°–120°W, 5°N–5°S) for the ENSO teleconnection (Trenberth, 1997; Trenberth and Stepaniak, 2001) and Tropical North Atlantic (TNA; 55°–15°W, 5°–25°N) for the TNA teleconnection (Enfield *et al*., 1999). TNA is selected partly because of its relatively strong association with Central American rainfall in the early wet season (Spence *et al*., 2004; Alfaro, 2007; Alfaro *et al*., 2016a; Maldonado *et al*., 2017), a time of year when some GCMs have had relatively lower skill (e.g., Kowal *et al*., 2021) to see how this SST zone could potentially enhance their skill. The indirect forecasts are constructed in pyCPT like the direct rainfall forecasts, but SST is selected as the predictor for either the Niño3.4 or the TNA region and then the same CCA method is used to transform the raw 1° GCM forecasts to 0.25° and statistically relate the SST predictors from a selected zone (e.g., Niño3.4) in a given period (e.g., MJJ) to rainfall over Central America in that period (e.g., MJJ).

For the statistical forecasts we run a linear regression of the observed rainfall against the spatial average of observed lagged (2 months prior) SST in the Niño3.4 region, the TNA region, and using both regions as predictor variables. This process is done for each season individually over the entire overlapping period of available CHIRPS/OISSTv2 data through 2016 (1982–2016), giving the statistical models over 30 years for training. The predictions are created using a cross‐validation leave‐one‐out‐approach in R (Kuhn, 2022) as described in Mason and Baddour (2008). For example, to predict rainfall for 1993 with the Niño3.4 statistical model, observed rainfall estimates in MJJ are regressed against spatially averaged observed March Niño3.4 SST across 1982–2016 (without 1993), setting 1993 as the prediction year. We select observed SST data 2 months prior to a forecast period to simulate operational forecasting conditions—because the GCMs are initialized on the first of the month (e.g., 1st of April), they do not benefit from the entirety of that month's data for the upcoming season (e.g., MJJ), so the observed SST values selected for the statistical forecast of MJJ rainfall, for instance, are averaged over March instead of April to not give the statistical model extra information the GCMs would not have had.

### 2.4 Evaluation criteria

For this assessment, we focus on the models' discrimination between tercile categories of rainfall to understand the models as they are deployed today by the CA‐COF. We also assess the models' identification rate of rainfall extremes above the 90th percentile and below the 10th percentile to better understand their potential for extreme event detection. We select the two alternative forced choice (2AFC) metric to evaluate tercile forecasts, and the Heidke skill score (HSS; Heidke, 1926) for low‐ and high‐rainfall detection (see Table 2). 2AFC assesses forecast ability to discriminate between categories and is preferred in part because it is an equitable metric across ordinal categories (treats all random forecasts with the same score; Mason and Weigel, 2009), is relatively common (e.g., Alfaro *et al*., 2018), and has been identified as a good metric to support decision‐making criteria (Weigel and Mason, 2011). HSS is preferred for extreme event detection because the metric is equitable (Hogan *et al*., 2010), relatively common (e.g., Higgins *et al*., 2004; Becker *et al*., 2012; Walker *et al*., 2019), and simple to understand (0 = skill of a random forecast, positive is better). Before the skill is tested, all the model and observational estimates are transformed into standardized anomalies by season across the hindcast period. For the MJJ period for instance, the mean and standard deviation of the MJJ forecasts from each model are calculated over 1993–2016 and then each estimate is subtracted from the mean and that value is divided by the standard deviation (as done in pyCPT).

**TABLE 2:** Summary of evaluation metrics with their relevant formulas

Metric	Summary	Formula
Heidke skill score (HSS)	Skill of categorical event detection (1 = perfect, 0 = skill of random forecast) (Heidke, 1926)	$\mathrm{HSS}=\frac{a+d-a_{r}-d_{r}}{n-a_{r}-d_{r}}$ *a*_*r*_ = (*a* + *b*)(*a* + *c*)/*n* *d*_*r*_ = (*b* + *d*)(*c* + *d*)/*n* *a* = Hits *b* = False alarms *c* = Misses *d* = Correct rejections Computed with “verification” package in R
Two‐alternative forced choice (2AFC)	Discrimination between events, i.e., below normal, normal, above normal (1 = perfect, 0.5 = skill of random forecast) (Weigel and Mason, 2011)	$p2\mathrm{AFC}=\frac{\sum_{i=1}^{m_{f-1}}\sum_{j=i+1}^{m_{f}}n_{0,i}n_{1,j}+0.5\sum_{k=1}^{m_{f}}n_{0,k}n_{1,k}}{n_{0}n_{1}}$ *n*_0_ = nonevents *n*_1_ = events *m*_*f*_ = number of forecast categories *n*_0,*i*_ = *i*th forecast issued, event did not occur *n*_1,*i*_ = *j*th forecast, event did occur *n*_0,*k*_ = number of forecasts for category k, no event occurred *n*_1,*k*_ = number of forecasts for category k, event did occur Computed with “afc” R package (Weigel, 2017)
One‐sample signed rank Wilcoxon test	Nonparametric test if sample median is significantly greater than a known value (Wilcoxon, 1945; Mann and Whitney, 1947)	$\begin{array}{c} \text{Test statistic }\left(W_{1}\right)=\sum R_{d}^{+}\\ \mu W_{1}=\frac{N_{r}\left(N_{r}+1\right)}{4}\\ \sigma W_{1}=\sqrt{\frac{N_{r}\left(N_{r}+1\right)\left(2N_{r}+1\right)}{24}}\\ z\;\text{statistic}=\frac{W_{1}-\mu W_{1}}{\sigma W_{1}} \end{array}$ Null hypothesis (*H*_0_): *z* ≤ known value Alternative hypothesis (*H*_1_): *z* > known value $R_{d}^{+}$ = ranks corresponding to positive difference between sample and known value *N*_*r*_ = number of difference scores (sample – known value) not equal to zero Computed with “stats” package in base R (R Core Team, 2021)
Kruskall–Wallis test	Nonparametric comparison of variance to test if multiple samples come from the same distribution (Kruskal and Wallis, 1952)	$H\;\text{statistic}=\frac{12}{N\left(N+1\right)}\sum \frac{R_{i}^{2}}{n_{i}}-3\left(N+1\right)$ Test *H* statistic against critical chi‐square value for N‐l degrees of freedom Null hypothesis (*H*_0_): Population medians are equal (critical chi‐square value ≥ H) Alternative hypothesis (*H*_1_): Population medians are not equal (critical chi‐square value < *H*) Computed with “stats” package in base R (R Core Team, 2021)
Conover‐Iman post hoc test	Pairwise comparisons following rejection of null hypothesis in Kruskall–Wallis test (Conover and Iman, 1979; Conover, 1999)	$\begin{array}{l} \text{Conover test statistic}\;\left(t\right)=\left|{\bar{R}}_{i}-{\bar{R}}_{j}\right|\;>\;t_{N-k}\sqrt{s^{2}\left[\frac{N-1-{\hat{H}}^{*}}{N-k}\right]}\left[\frac{1}{n_{i}}+\frac{1}{n_{j}}\right]\\ {\hat{H}}^{*}\left(\mathrm{tie}\;\text{corrected statstic of}\;\mathrm{KW}\;\text{test}\;\right)=\frac{\frac{12}{N\left(N+1\right)}\sum_{i=1}^{k}\frac{R_{i}^{2}}{n_{i}}-3\left(N+1\right)}{1-\frac{\sum_{i=1}^{r}\left(t_{i}^{3}-t_{i}\right)}{N^{3}-N}}\\ s^{2}=\frac{1}{N-1}\left[\sum R_{i}^{2}-N\frac{{\left(N+1\right)}^{2}}{4}\right] \end{array}$ Null hypothesis (*H*_0_): Population medians are equal Alternative hypothesis (*H*_1_): Population medians are not equal *k* = number of groups *N* = total number of samples *n*_*i*_ = sample number in the *i*th group *R*_*i*_ = total sum of ranks in *i*th group Computed with “PMCMRplus” and “rcompanion” R packages (Mangiafico, 2022; Pohlert, 2022)

To assess comparative performance for each season and subregion, we use two nonparametric statistical tests: a one‐sample signed rank Wilcoxon test (Wilcoxon, 1945) and a Kruskal–Wallis test (Kruskal and Wallis, 1952) combined with post hoc testing using the Conover‐Iman method (Conover and Iman, 1979; Conover, 1999), which is less well‐known than Dunn but has more statistical power (Gilbert, 2019). We select nonparametric tests instead of their parametric counterparts (*T* test and ANOVA) because the data sample sizes are limited and we do not assume normality or homogeneity of variance within each ensemble spread. To answer our first comparative question—do the ensembles significantly outperform the statistical models or a random forecast, we use the Wilcoxon test. For this test we first identify the skill of the top performing statistical model against the observations and check whether that model is better than a random forecast for a given period/subregion (e.g., North Pacific in MJJ). We then set the best performing statistical model skill as the value to beat (or use the skill of a random forecast if all the statistical models perform worse than a random forecast) to see if the skill of the ensemble spread against the observations is significantly different from the best statistical model skill.

To compare across distributions of ensembles, that is, C3S vs. NMME, we use a Kruskal–Wallis test to assess if there are any significant differences between the skill of the spread of the ensembles for a given seasonal period and subregion. Then we conduct post hoc testing using Conover when the Kruskal–Wallis test shows there are significant differences warranting pair‐wise comparisons between groups to identify top performers. Because we are only interested in identifying the top direct and indirect forecasts, we only perform Kruskal–Wallis/post hoc testing to compare the ensembles that are found to be significantly better than the best statistical forecasts and a random forecast. We then assess differences in predictability between the ensembles by evaluating the prediction skill of SST in known teleconnection regions using Pearson's *R*. The SST locations of interest include both the Niño3.4 region and the TNA region. We also test average association (using Pearson's *R*) between observed SST and rainfall over Central America over different wet season periods to compare with the SST forecast skill.

## 3 RESULTS

### 3.1 Skill of discrimination between above normal, normal, and below normal rainfall categories

**FIGURE 3:**
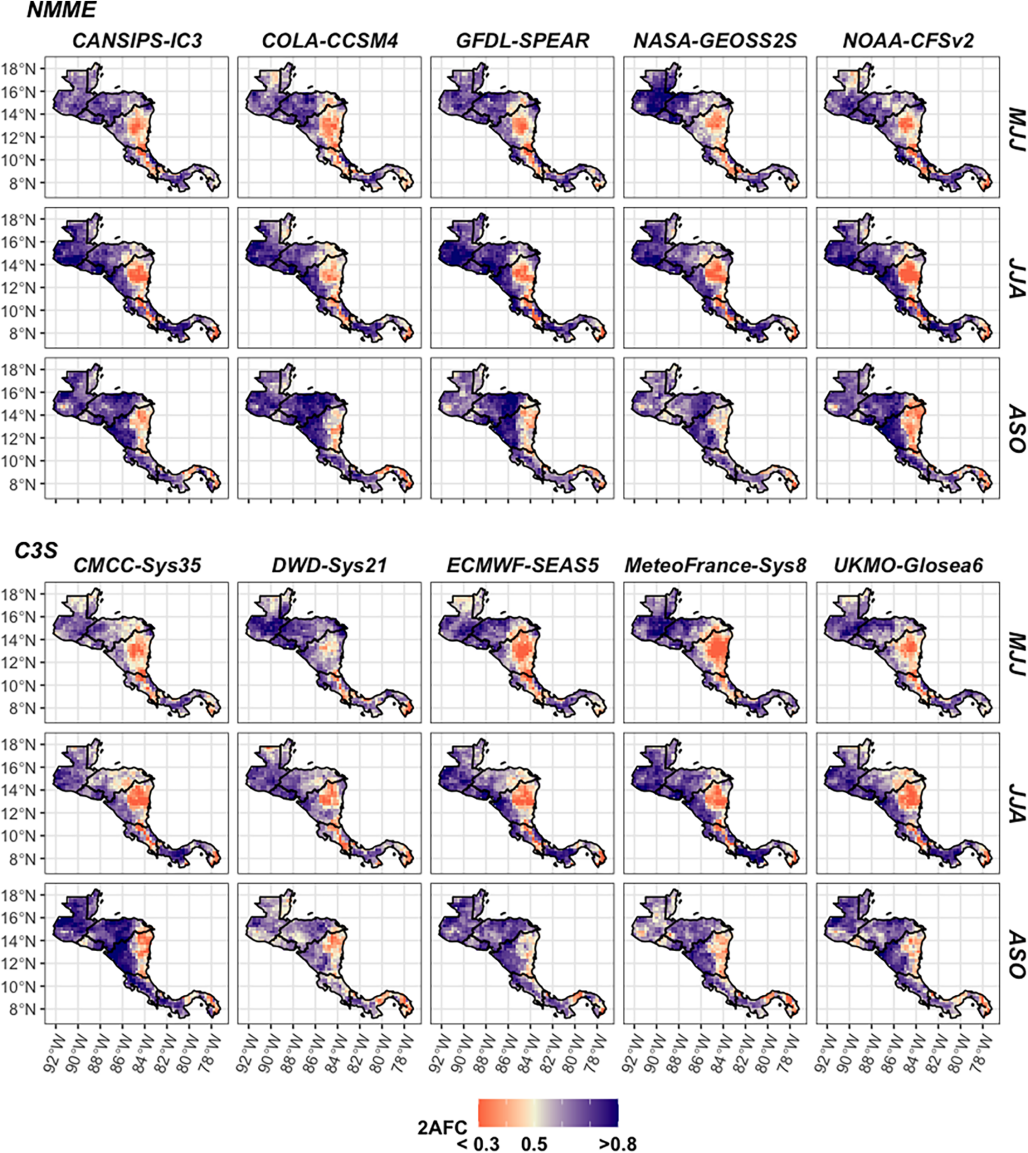
Rainfall skill for direct tercile forecasts using 2AFC across individual models in the wet season. 0.5 is equal to the skill of using a random forecast. [Colour figure can be viewed at wileyonlinelibrary.com]

**FIGURE 4:**
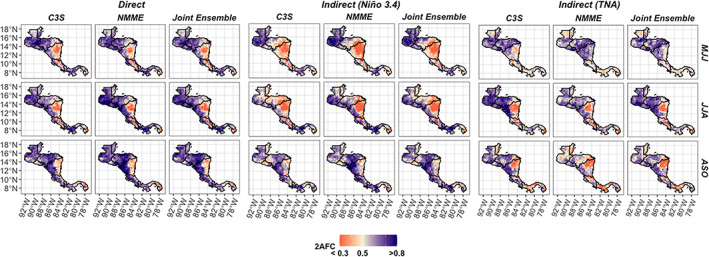
Rainfall skill of the ensemble means using C3S, NMME, and the Joint Ensemble. All models in the study are compared using their direct and hybrid forecasts with 2AFC skill. [Colour figure can be viewed at wileyonlinelibrary.com]

The 10 GCMs assessed show a similar seasonal and geographic variation in skill over Central America when forecasting rainfall directly (Figure 3). For instance, in eastern Nicaragua, the direct forecasts from the individual models have relatively low skill compared to the rest of the isthmus, while skill is often higher along the Pacific coast (Figure 3). Combining the models into ensembles also shows similar variation in seasonal/geographic skill variability, and the ensembles tend to perform similarly within a given method (i.e., direct or Niño3.4/TNA indirect; Figure 4). Eastern Nicaragua is still a relatively lower skill zone, and the North Pacific is an area with high skill (Figure 4). Looking across forecasting methods (e.g., direct vs. Niño3.4 indirect), the direct rainfall forecasts showcase higher skill than the indirect forecast skill in most cases except for the early wet season (MJJ) when the indirect forecasts demonstrate better performance compared to the direct forecasts in multiple locations (Figure 4, top row). The TNA indirect forecasts (Figure 4, right group), for instance, have relatively higher skill compared to other forecast methods (Niño3.4 indirect and direct) over eastern Nicaragua in MJJ, and the Niño3.4 indirect forecasts have highest skill in Guatemala during this period (Figure 4, middle group). While the direct ensemble skill tends to be highest in most other cases (Figure 4, left group), the Niño3.4 indirect forecasts also have similar skill in the late wet season (ASO; Figure 4, bottom row).

**FIGURE 5:**
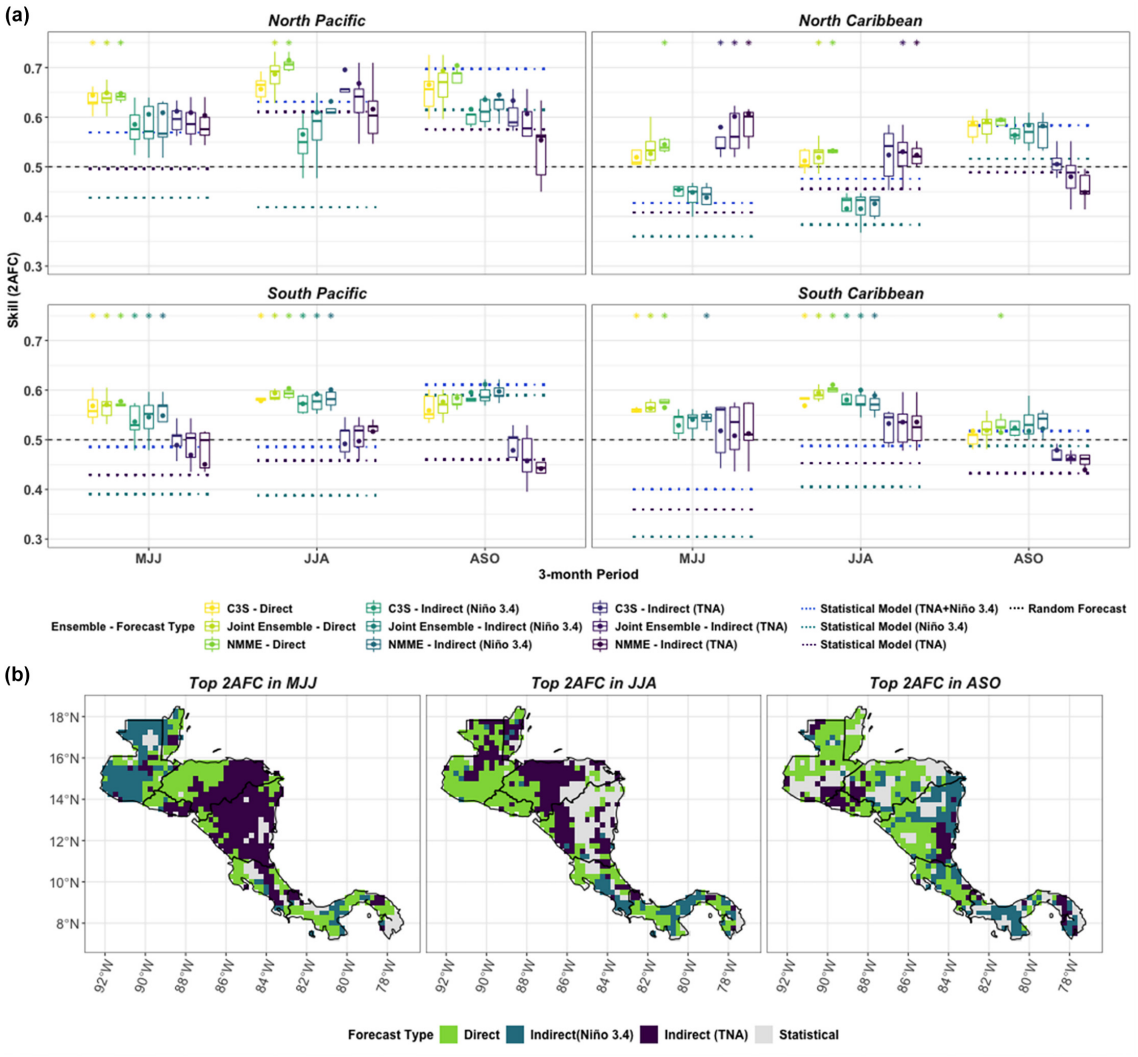
(a) Spatially averaged comparison of forecast skill using 2AFC across seasons and grouped by subregion as defined in Figure 1a. Boxplots are catogorized by ensemble and show the spread of the individual model means contributing to each ensemble. Dots within boxplots show the skill of the ensemble mean across all contributing models using 2AFC. Dashed lines refer to statistical models (except 0.5 equal to skill of a random forecast). Top performing direct and indirect forecasts are given stars (above the relevant boxplot), which indicate if forecasts meet three criteria: they are significantly better than a random forecast, significantly better than the best statistical forecast, and are not significantly worse than another ensemble, e.g., an ensemble that beats a random and statistical forecast but is significantly worse than another ensemble will not receive a star. (b) Top performing forecasting approach using the 2AFC score grouped by season. [Colour figure can be viewed at wileyonlinelibrary.com]

The visual assessments of absolute skill (Figures 3 and 4) are confirmed when statistically testing the differences between the spatially averaged skill by subregion (Figure 5a) and tagging the top performer by location for each seasonal period (Figure 5b). On average, the North Pacific is the highest skill zone with highest skill occurring in JJA (Figure 5a, top left panel). The differences in spatially averaged skill are smaller between the other sub regions, but similar differences still exist—e.g., direct forecasts have the lowest skill in the North Caribbean of all the subregions in JJA (Figure 5a, top right panel), which is mirrored in the absolute skill plots of the direct forecasts (Figure 3). Between the direct and indirect ensembles, the direct forecasts also tend to have one of the highest skill scores in most cases, especially in the North Pacific (Figure 5a, top left panel). Similar exceptions persist. For instance, a TNA indirect forecast tends to have the highest skill in MJJ in the North Caribbean (Figure 5a, top right panel and Figure 5b, left panel). Although the spatially averaged skill of the Niño3.4 indirect forecasts are not best in the North Pacific generally in the early wet season (MJJ) (Figure 5a, top left panel), the Niño3.4 indirect forecast still demonstrates more targeted value over Guatemala as the top performer in MJJ (Figure 5b, left panel). The direct and Niño3.4 indirect forecasts also tend to have the highest skill in the South Pacific and South Caribbean, often showing similar skill in ASO (Figure 5a, bottom two panels).

The direct and indirect forecasts do not consistently outperform the statistical forecasts though for all periods. The forecasts are often better than a statistical forecast based on one teleconnection region alone (e.g., Niño3.4 or TNA), but the combined statistical model skill is usually the best performer of the statistical models, with peak skill in the late wet season (Figure 5a). In the North Pacific, for instance, the ensembles are better than the statistical forecasts in the early to middle wet season (MJJ/JJA), but their skill is not significantly better than using a combined statistical forecast based on TNA and Niño3.4 SST in ASO (Figure 5a, top left panel). Although direct forecast skill is high in the North Pacific in ASO, the comparative value of using the direct forecasts over this subregion is clearer in the early to middle wet season (MJJ and JJA) when the statistical model skill is lower.

### 3.2 Skill of high‐ and low‐rainfall extremes

**FIGURE 6:**
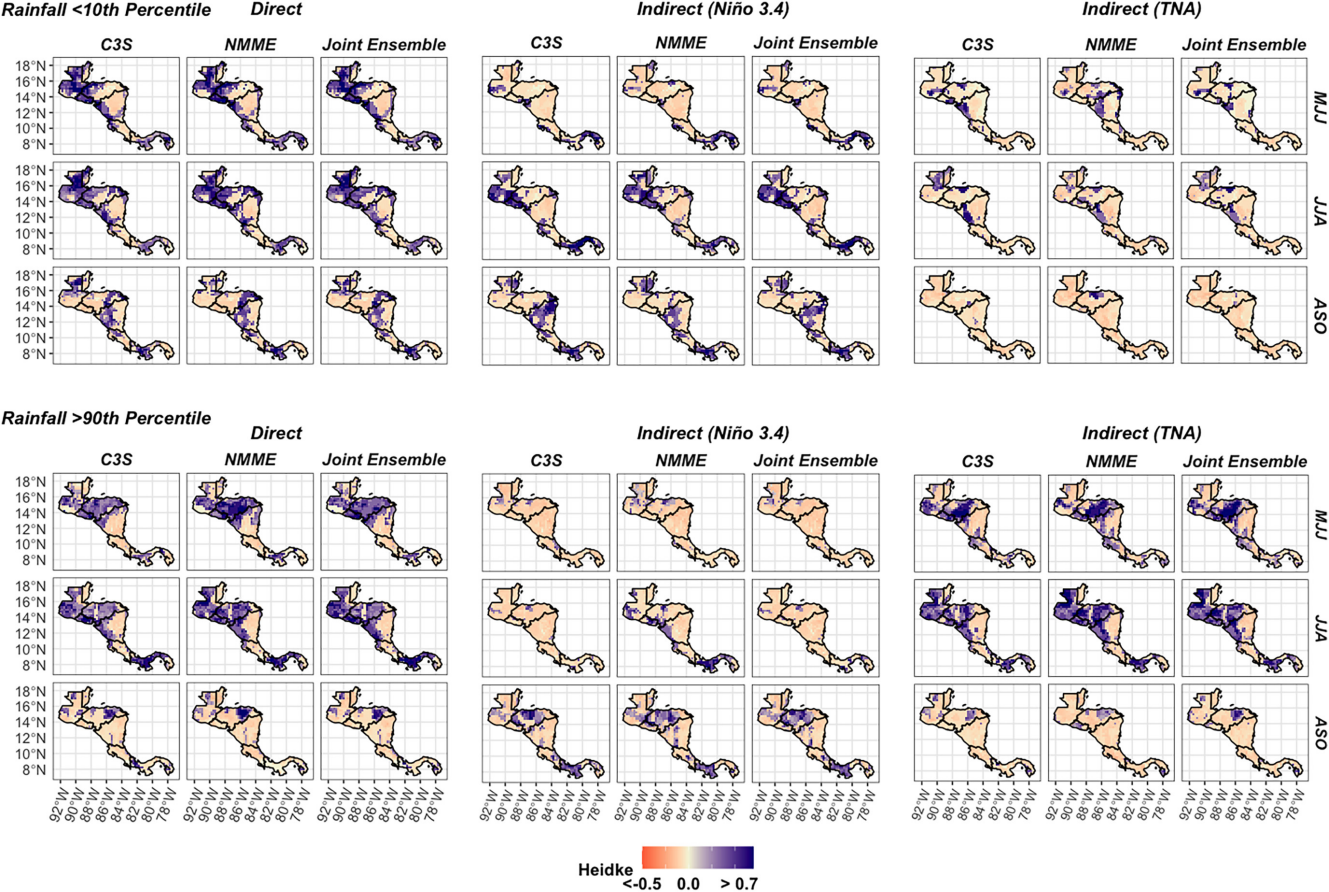
Ensemble mean skill for high‐ and low‐rainfall extremes using HSS, where 0.0 is equal to the skill of a random forecast. Skill is plotted over separate parts of the wet season—MJJ, JJA, and ASO. [Colour figure can be viewed at wileyonlinelibrary.com]

Ensemble skill is more limited when the models are used to predict extreme rainfall (Figure 6 and see Figure S3 for skill of direct forecasts of rainfall extremes plotted individually by model). In the late wet season (ASO), for instance, skill is almost always zero (Figure 6, bottom row of Rainfall <10th percentile and Rainfall >90th percentile groups). Eastern Nicaragua is still a trouble spot for the direct and indirect forecasts (Figure 6), but direct forecast skill is still high in much of the North Pacific during the early to middle wet season (MJJ/JJA; Figure 6, left group). When comparing the absolute skill of direct and indirect extreme rainfall forecasts, the skill also does not have the same seasonal patterns as for tercile discrimination, emphasizing how the application matters when evaluating forecasting methods. For instance, although a direct forecast tends to have high spatial skill for the detection of low‐ and high‐rainfall extremes in the early to middle wet season (MJJ/JJA; Figure 6, left group), a TNA indirect forecast has higher skill in some areas of Central America for detection of high‐rainfall extremes in JJA (Figure 6, bottom right group), a period when direct forecasts often performed better for tercile rainfall discrimination (Figure 5a).

**FIGURE 7:**
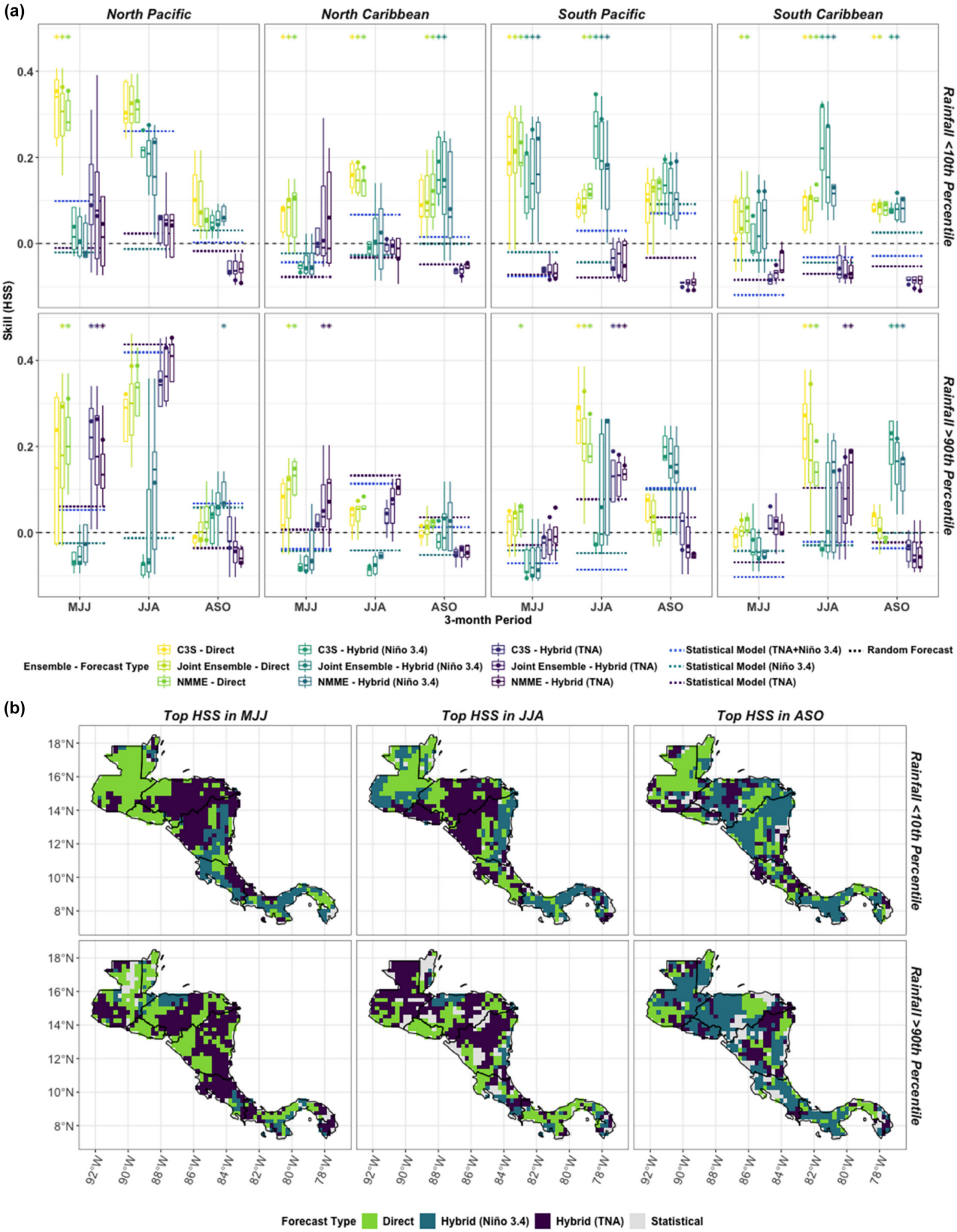
(a) Spatially averaged comparison of forecast skill for low‐ and high‐rainfall extremes using Heidke (HSS) across seasons and grouped by subregion. Boxplots are catogorized by ensemble and show the spread of the individual model means contributing to each ensemble. Dots within boxplots show the skill of the ensemble mean across all contributing models using HSS. Dashed lines refer to statistical models (except 0.0, equal to skill of a random forecast). Top performing direct and indirect forecasts are given stars, which indicate if forecasts meet three criteria: They are significantly better than a random forecast, significantly better than the best statistical forecast, and are not significantly worse than another ensemble, e.g., an ensemble that beats a random and statistical forecast but is significantly worse than another ensemble will not receive a star. (b) Top performing forecasting approach using HSS grouped by season. [Colour figure can be viewed at wileyonlinelibrary.com]

The skill of the ensemble means is similar within forecasting methods (Figure 6), and the differences between the two ensembles are often not significant when they are both top performers (Figure 7a, e.g., both C3S and NMME direct forecasts get stars in most cases when a direct method performs well), meaning method choice matters more than ensemble choice for extreme rainfall detection. The skill of extreme rainfall detection has higher variability within ensembles than for discrimination between rainfall terciles though, which makes it less easy to identify a top performing forecasting method for this application. The ensemble mean often outperforms the median of the contributing models (Figure 7a), but not as consistently as when the ensemble mean is used to discriminate between terciles (Figure 5a). The C3S skill variations exemplify this variability. The median of the C3S ensemble is sometimes higher than the NMME mean (e.g., MJJ in South Pacific; Figure 7a, top panel second from right), the top of the interquartile range of the C3S ensemble is often higher than the top of the interquartile range of the NMME for low‐ and high‐rainfall extremes even when the mean is lower (e.g., MJJ in North Pacific; Figure 7a, left panels), and the interquartile spread of C3S is often larger than NMME, meaning some C3S contributors drag down the score of the C3S mean while others perform much better (Figure 7a).

When compared to the statistical models, the direct and Niño3.4 indirect forecasts are often significantly better at detecting low‐rainfall extremes (Figure 7a, top panels). The TNA indirect models show significantly better skill than the statistical models in multiple early and middle wet season periods (MJJ/JJA) when detecting high‐rainfall extremes but are never a standalone method that is significantly better than the others (any time a TNA indirect forecast gets a star, another forecast method also receives a star; Figure 7a, bottom panels). Generally direct and indirect methods showcase more promise for detections of low‐ over high‐rainfall extremes—the ensembles significantly outperform the statistical forecasts more often when detecting low‐rainfall extremes as compared to high‐rainfall extremes (Figure 7a); and the top performing forecast type has more spatial consistency for low‐rainfall extremes (Figure 7b, e.g., direct forecasts over Guatemala in MJJ).

### 3.3 Predictive skill of key teleconnection regions: ENSO and TNA

**FIGURE 8:**
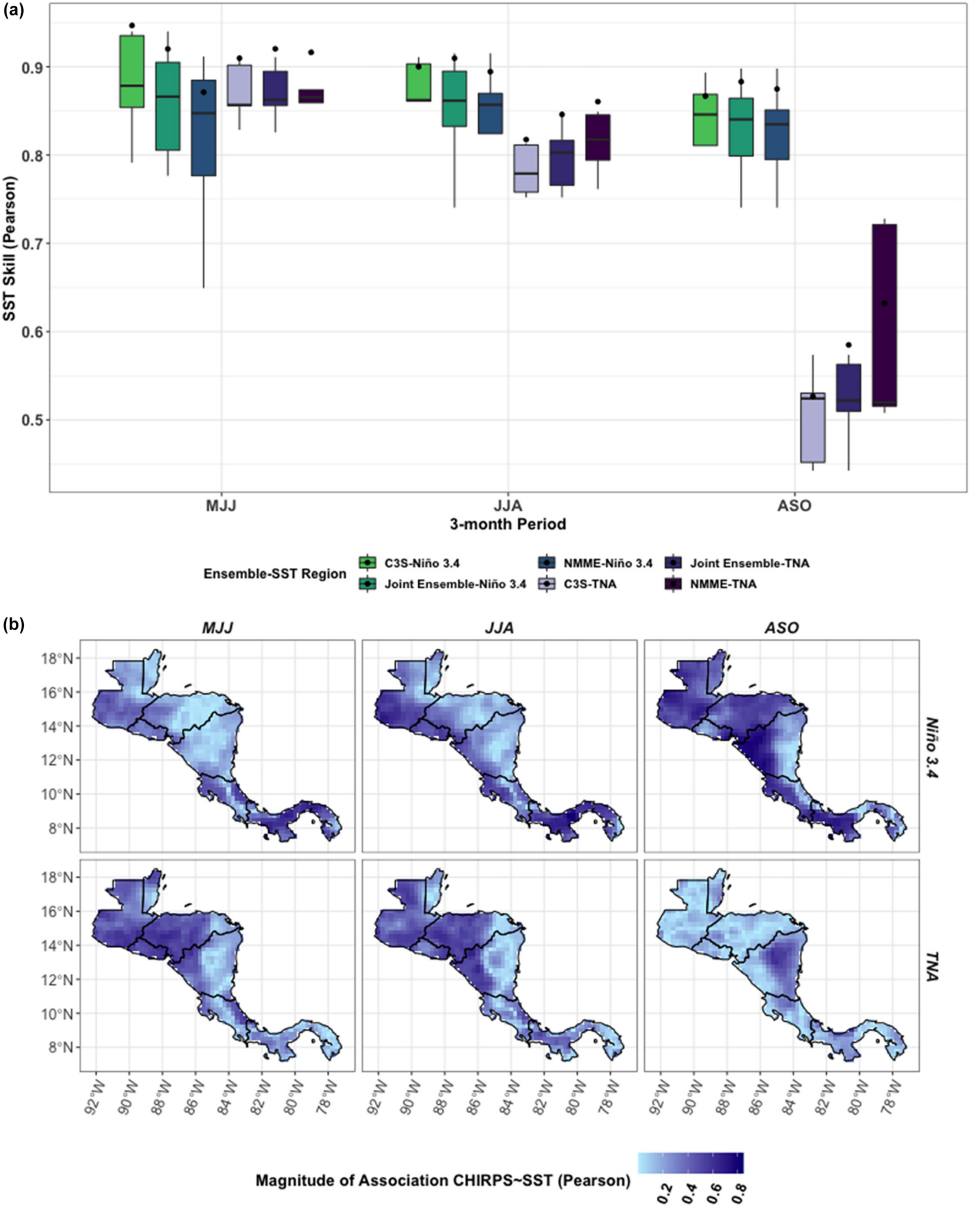
(a) Ensemble SST skill measured using Pearson's R, plotted by season for the Niño3.4 and TNA SST regions. Boxplots represent the range of contributing models; points refer to the skill of each ensemble's mean. (b) Pearson's R between observed rainfall and SST in the Niño3.4 and TNA regions, plotted by season [Colour figure can be viewed at wileyonlinelibrary.com]

The skill of the ensembles' predictions of SST over the Niño3.4 region is consistently high. The association between each model and the observed Niño3.4 SST exceeds *R* = 0.7 for most models in the wet season periods, and the association of the ensemble means are between 0.85 and 0.98 for Niño3.4 (Figure 8a). Differences in SST skill over Niño3.4 therefore are not likely driving the differences in forecast skill over the region. SST skill is lower over the TNA region in the late wet season (Figure 8a), but this is also when the TNA region is least associated with rainfall, compared to the earlier months (Figure 8b, bottom right panel). The stronger performance of the TNA indirect forecasts correlates with this association between TNA SST and Central American rainfall, as the TNA indirect forecasts tend to perform better earlier in the wet season (Figures 4, 5, 6, 7). The relative strength of the associations between each SST zone and regional rainfall also somewhat aligns with the differences in rainfall skill. In the North Caribbean, for instance, the association between Niño3.4 (TNA) SST and rainfall is more than 15% (20%) lower than in the North Pacific in the early to middle wet season (Figure 8b).

## 4 DISCUSSION

No single model or method outperformed the others in all cases. Choosing between forecasting approaches is not quite as simple as picking a “winner,” but our evaluation highlighted a few comparisons that could be useful for decision making, and act as a starting point to tailor seasonal forecasts for use cases across the region:

1. ***The North Pacific i s a relatively high ‐ skill zone** **for seasonal rainfall*** ***forecasts*.** This is true not only for tercile discrimination across the wet season but for low‐ and high‐rainfall extremes detection in the early and middle wet season (MJJ/JJA). Highest skill occurs in JJA in the North Pacific for both tercile discrimination and rainfall extremes detection, which is when the most distinct and consistent mid‐summer dry period occurs of all the subregions (see Figure 1a). These findings have useful implications for drought preparedness. The CADC (a drought hotspot; Gotlieb *et al*., 2019) is in the North Pacific, and high skill for low‐rainfall extremes detection could potentially help mitigate drought impacts in this region. High skill in the North Pacific in the early wet season is also important for the first planting period of the wet season. For corn and bean crops in Guatemala, for instance, planting occurs primarily in the first 10 days of May and June (INSIVUMEH, 2022a), underscoring the importance of the MJJ forecasts for communicating rainfall information to farmers at the local technical agroclimatic forums (INSIVUMEH, 2022b).
2. ***Trouble spots for seasonal rainfall forecasts include eastern Nicaragua for most applications*** **,** ***and the late wet season for low‐ and high‐rainfall extremes detection***. TNA indirect forecasts are relatively better than other forecasting methods over eastern Nicaragua during the early wet season (MJJ) for tercile rainfall detection, but no forecasting method has high skill when detecting high‐ or low‐rainfall extremes over eastern Nicaragua. Lower skill over this area may be a result of limited gauges over the Caribbean slope as compared to the Pacific slope (e.g., Maldonado *et al*., 2016), and although similar spatial variability in skill was seen against other gridded observed datasets that use multiple inputs such as satellites and gauges (Figure S2), it would be worth further exploring the spatial skill differences using gauge data alone in future studies. Limited skill across Central America in the late wet season for detection of high‐rainfall extremes is unfortunate but unsurprising, as this period is when high‐rainfall extremes most often occur over Central America, primarily on the Pacific slope (Taylor and Alfaro, 2005; Maldonado *et al*., 2013). High variability of rainfall during the late wet season is compounded by the hurricane season, which occurs in October, making rainfall more difficult to predict. This kind of variability is seen in the TNA SST skill (Figure 8a), which drops significantly in ASO, when storms are forming over the Atlantic.
3. ***We saw larger differences betwe en forecasting methods (e.g*** **.,** ***direct vs. indirect) than between ensemble types (C3S vs. NMME)*** **.** Similarities in skill across ensembles simplify decision making, as the ensemble choice matters less than picking a forecasting method for a given location/season. Why the dynamically driven forecasts (direct and indirect) are so similar within a forecasting method could arise from a variety of reasons, including limited observational data that constrains how scientists understand the atmospheric processes over a given location, regional predictability characteristics (e.g., eastern Nicaragua may be harder to predict because the rainfall could be more determined by atmospheric variability than oceanic influences, which are easier to simulate), or due to the nature of the models themselves including structural and initialization choices, but the exact reasoning behind the similarities lies outside the scope of this study. The similarities are unlikely due to observational reference choice, however, as the similarities persist across multiple observational datasets (see Supporting Information).
4. ***The comparative advantage of using direct*** **,** ***indirect*** **,** ***and statistical methods varies by application*** **.**

- **Tercile rainfall discrimination:** Direct and indirect forecasts are relatively better than statistical forecasts in the early to middle wet season (MJJ/JJA) as compared to the late wet season (ASO) when statistical skill is often high. Indirect forecasts have comparative advantages in three primary locations: a Niño3.4 indirect forecast in Guatemala in the early wet season (MJJ), a TNA indirect forecast over eastern Nicaragua in the early wet season (MJJ), and a TNA indirect forecast centred over the 86^0^ W longitude line in the early to middle wet season (MJJ/JJA).
- **Low‐rainfall extremes detection:** Direct and Niño3.4 indirect forecasts significantly outperform statistical models in most cases, but in northern Central America in the early and middle wet season (MJJ/JJA), a TNA indirect forecast is the top performer.
- **High‐rainfall extremes detection:** High‐rainfall extremes are more difficult for the models to detect than low‐rainfall extremes across the wet season. Top performers are less spatially and seasonally consistent, and the late wet season (ASO) is consistently a low‐skill period.

The exact nature of the relationship between forecast skill and climatic drivers is still unclear. Seasonal differences in SST skill are unlikely to drive differences in rainfall skill, as SST skill is often high across the models, especially when SST for a given zone (e.g., Niño3.4) is highly correlated with rainfall in Central America. The GCMs are relatively good at predicting Niño3.4 SST (Figure 8a), which aligns with findings from other studies (e.g., Zhang *et al*., 2017). SST skill does drop over TNA in the late wet season, but rainfall also has a weaker relationship with SST in the TNA region during ASO compared to earlier in the wet season (Figure 8b), so lower TNA SST skill may have a negligible effect on the skill of the rainfall predictions. The seasonal patterns of Niño3.4 and TNA SST associations with rainfall also align with other studies, which show ENSO has a stronger relationship with rainfall in the late wet season (e.g., Waylen and Quesada, 2001; Spence *et al*., 2004), and TNA has a strong association with rainfall in the early wet season (Spence *et al*., 2004; Alfaro, 2007; Maldonado *et al*., 2017). The comparative value of using dynamically driven forecasts in the early to middle wet season (MJJ/JJA) in the North Pacific is possibly due to the strong relationship between ENSO and regional rainfall during the late wet season, which increases the skill of the statistical forecasts (Figure 8b).

The regional variations in the strength of SST association with rainfall may also be related to rainfall forecast skill. In the Caribbean and the southern part of the isthmus, all forecast skill tends to be lower, with an over 10% drop in 2AFC and HSS scores for normal and extreme rainfall compared to their skill in the North Pacific (see Figures 5 and 7). SST associations with rainfall are also strongest in the North Pacific across much of the wet season (Figure 8b). As ENSO and TNA are not the only factors affecting rainfall over Central America, other influences are important to consider for improving rainfall predictions, especially in low‐skill zones (e.g., ITCZ migration, tropical cyclone formation; Hidalgo *et al*., 2015; Maldonado *et al*., 2018; Durán‐Quesada *et al*., 2020). The majority of eastern Nicaragua, for instance, has relatively low influence from the Pacific Ocean as compared to the rainfall in the North Pacific (Figure 8b) and may have stronger relationships with other drivers. For instance, the CLLJ branches around 10^0^ N and travels north along Nicaragua and south across the southern isthmus (Hidalgo *et al*., 2015), and the models' abilities to capture this process and how it interplays with the Cordillera (since mountainous terrain is often a challenge for numerical weather prediction; e.g., Serafin *et al*., 2018) could play a significant role in rainfall forecast skill, which would be worth exploring in future work.

This study aimed to enhance the comparisons of C3S and NMME by including some basic statistical modelling for reference. The statistical models generated here show relatively high forecast skill and would be worth exploring further to see how their skill could be improved. For instance, two main drivers of rainfall were selected based on the literature, but predicting other drivers of rainfall (e.g., windspeed) or conducting an empirical analysis to identify potential hidden drivers of rainfall beyond traditional indices could further improve on the indirect and statistical modelling approaches (e.g., Kretschmer *et al*., 2016; Kretschmer *et al*., 2017; Alfaro *et al*., 2018; di Capua *et al*., 2019; Renard and Thyer, 2019). Other study limitations include the limited time period of the hindcasts (1993–2016), which increases the uncertainty of a skill assessment (Tippett *et al*., 2005; Shi *et al*., 2015). As more models become publicly available for longer periods, it would be worth reconducting the analysis to see how top performers might change over the region. Furthermore, this work demonstrated spatial and seasonal variations in skill that are worth exploring further to understand how different processes may affect model skill. SST skill is relatively consistent across the GCMs (Figure 8a), but there is a need for further study of how they capture atmospheric processes, to make sure the models are predicting rainfall for the right reasons (e.g., modulating moisture transport mechanisms like the CLLJ correctly). Next steps would include process‐based evaluations that examine other important variables like sea level pressure and wind speed that can affect moisture transport over Central America and test how the ability of the models to represent those process relates to the skill of their final outputs.

## 5 CONCLUSIONS

This study systematically compares seasonal rainfall forecasts from two multimodel ensembles relative to a statistical forecasting approach and examines different ways they could be deployed using direct and indirect forecasting techniques. Results demonstrate that both C3S and NMME have high skill when detecting below normal, normal, and above normal rainfall in the wet season, especially over the North Pacific region of Central America. Their comparative value is often highest in the early to middle wet season (MJJ/JJA) when the statistical forecast skill is relatively lower. Indirect forecasts can improve upon direct rainfall forecasts when skill is low, primarily using a TNA indirect forecast during the early wet season period in the North Caribbean. The multimodel ensembles also show promise for detecting low‐rainfall extremes in drought hot spots like the CADC but are less reliable for detecting high‐rainfall extremes in the North Pacific where most high‐rainfall extremes occur. The variability of top performing forecasting combinations demonstrates how multiple tools can help improve forecast skill rather than selecting one forecasting method or model as the “winner” for all cases. Continuing to explore drivers of skill over the region and identifying when and where to trust these forecasts is important to ensure they are as reliable as possible for seasonal rainfall planning.

## AUTHOR CONTRIBUTIONS

**Katherine Kowal:** Conceptualization; data curation; formal analysis; visualization; writing – original draft; writing – review and editing; project administration; investigation; methodology; funding acquisition; validation; software. **Louise Slater:** Conceptualization; writing – review and editing; supervision; methodology; visualization; resources. **Alan García López:** Conceptualization; formal analysis; methodology; data curation; writing – review and editing. **Anne F. Van Loon:** Conceptualization; methodology; supervision; writing – review and editing; resources.

## CONFLICT OF INTEREST

The authors declare no potential conflict of interest.

## Supporting information


**Data S1.** Supporting Information.

## Data Availability

The forecast data are publicly accessible via the Climate Data Store and the IRI data library. All reference data are publicly available as described in section 2.
