# Supplementary material for: A comparison of seasonal rainfall forecasts over Central America using dynamic and hybrid approaches from Copernicus Climate Change Service seasonal forecasting system and the North American Multimodel Ensemble
Source: Int J Climatol. 2023 Jan 6;43(5):2175–99. doi: 10.1002/joc.7969 (PMC11921286; doi:10.1002/joc.7969)
Supplement: Supplementary file 1 — Data S1. Supporting Information. [file JOC-43-2175-s001.docx]

## Supplementary Information

## S.1 Observational Data Analysis

We selected CHIRPS as the rainfall verification dataset for this study, but multiple observational datasets were considered for use in this evaluation. Of the variety considered, we compared rainfall at 0.25’ monthly resolution using CHIRPS (Funk et al., 2014), the Global Precipitation Climatology Centre (GPCC - Schneider et al., 2018), and version 2 of Multi-Source Weighted-Ensemble Precipitation (MSWEP - Beck et al., 2017), which we selected because they include a variety of data inputs and their temporal coverage all span 1993-2016. There is a range in their anomalies of total seasonal rainfall over the time series, and MSWEP tends to lie on the extreme ends of the rainfall anomalies (Figure S1). The range across the compared datasets, however, is often less than the spread of the models – on average less than one standard anomaly for the compared regions and seasons (Figure S1).


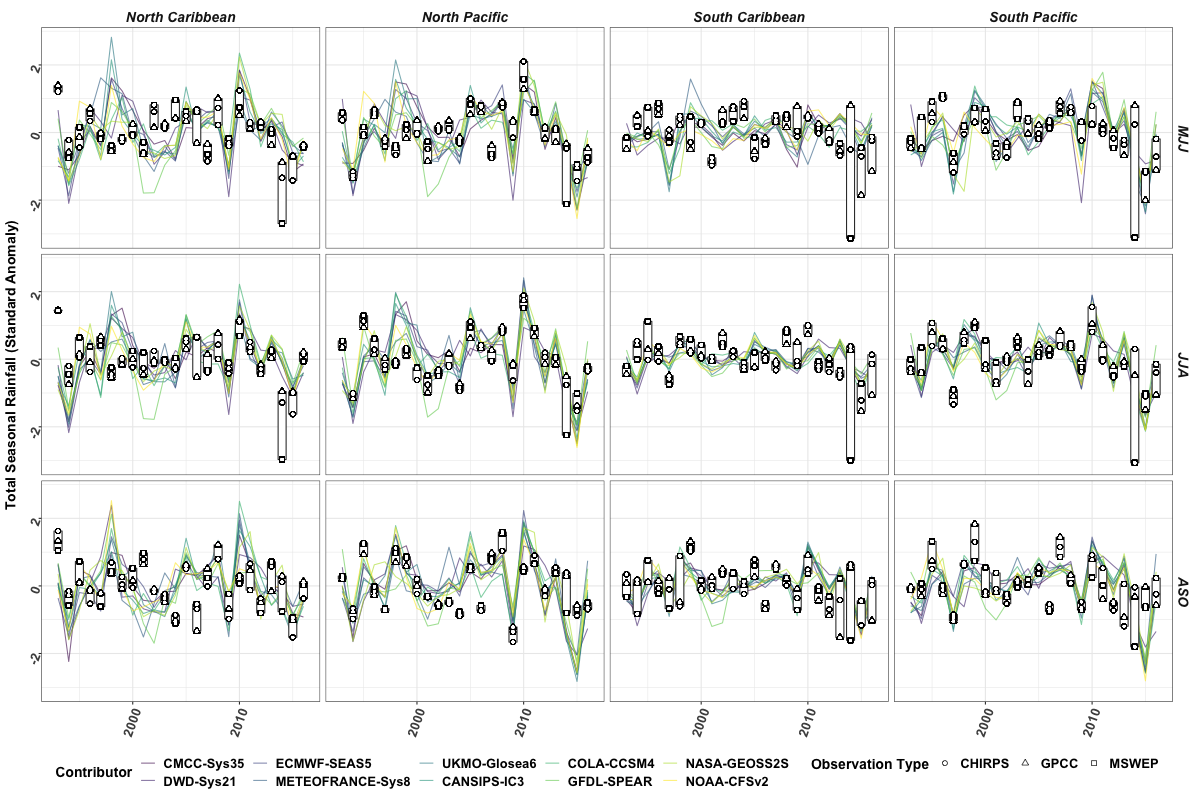


Figure S1. Comparison of observed datasets over 1993-2016 plotted as box plots and points against direct rainfall forecasts from the compared models for each spatially-averaged compared zone and season.

In terms of implications for model skill, the correlation between the observed data and model skill is similar across GPCC, MSWEP, and CHIRPS (Figure S2). The models perform similarly compared to each other for each of the observed datasets, often demonstrating lower association with the observed data over the North Caribbean in Nicaragua, and higher association over the North Pacific over each of the seasons compared.


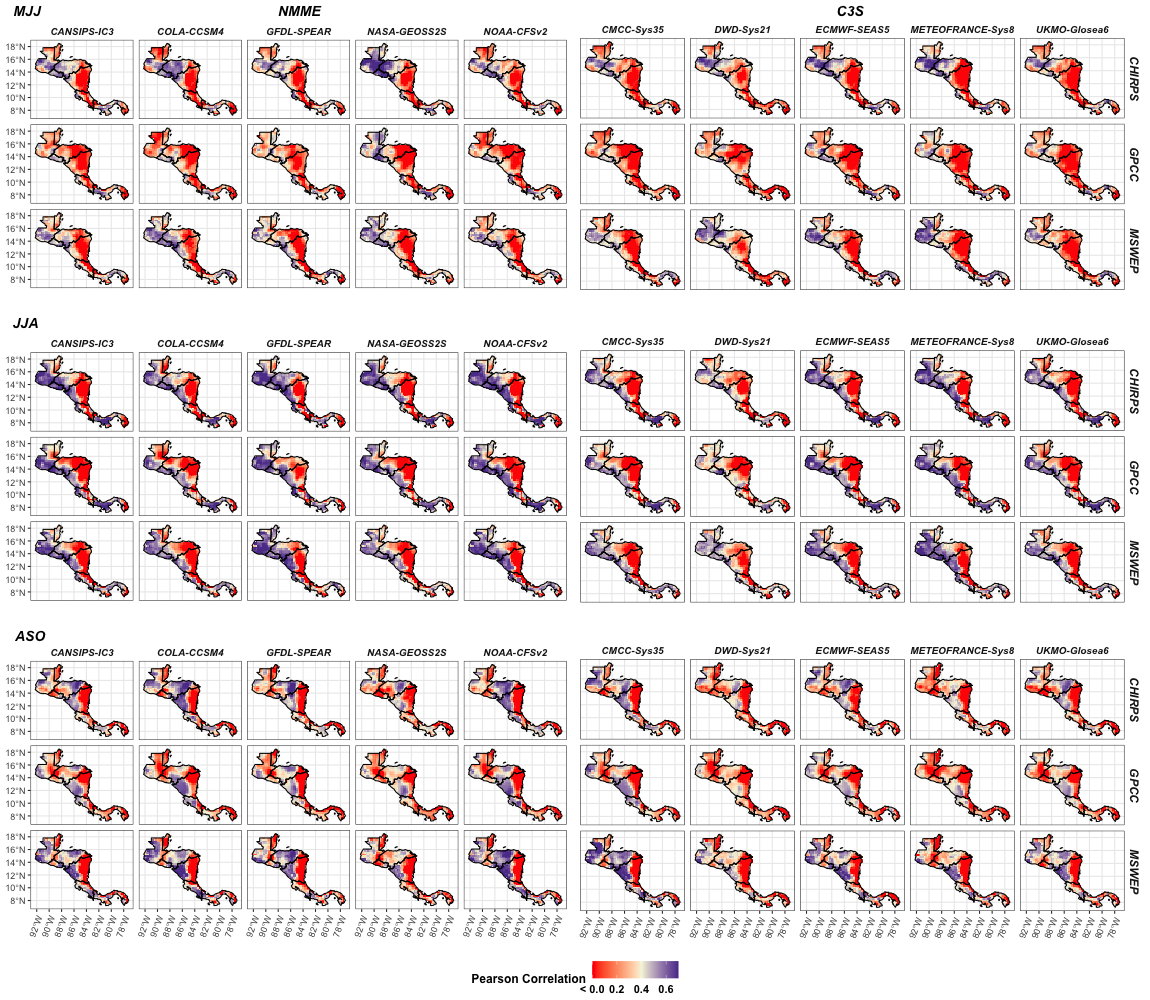


Figure S2 Association (Pearson’s R) is plotted spatially between direct model mean rainfall forecasts and observations from different observational data sets over May-July, June-August, and August-September.

## S.2 Individual Model Skill for Detecting Low and High Rainfall Extremes

Here we illustrate how individual model skill varies for detections of low and high rainfall extremes. There is more variability across models (Figure S3) than between ensembles (Figure 6) – as might be expected, but similar geographic and seasonal variations occur. For instance, the North Pacific is still a relatively high skill zone and eastern Nicaragua is a relatively low skill zone. The variability of the individual model plots illustrate why forecast skill within ensembles has a larger range for detections of extremes. For example, DWD (System 21) has almost very limited skill for detecting high rainfall extremes across the wet season, as compared to Meteofrance (System 8), which has high skill across most of the isthmus in the early to middle wet season (MJJ/JJA) (Figure S3 bottom right group).


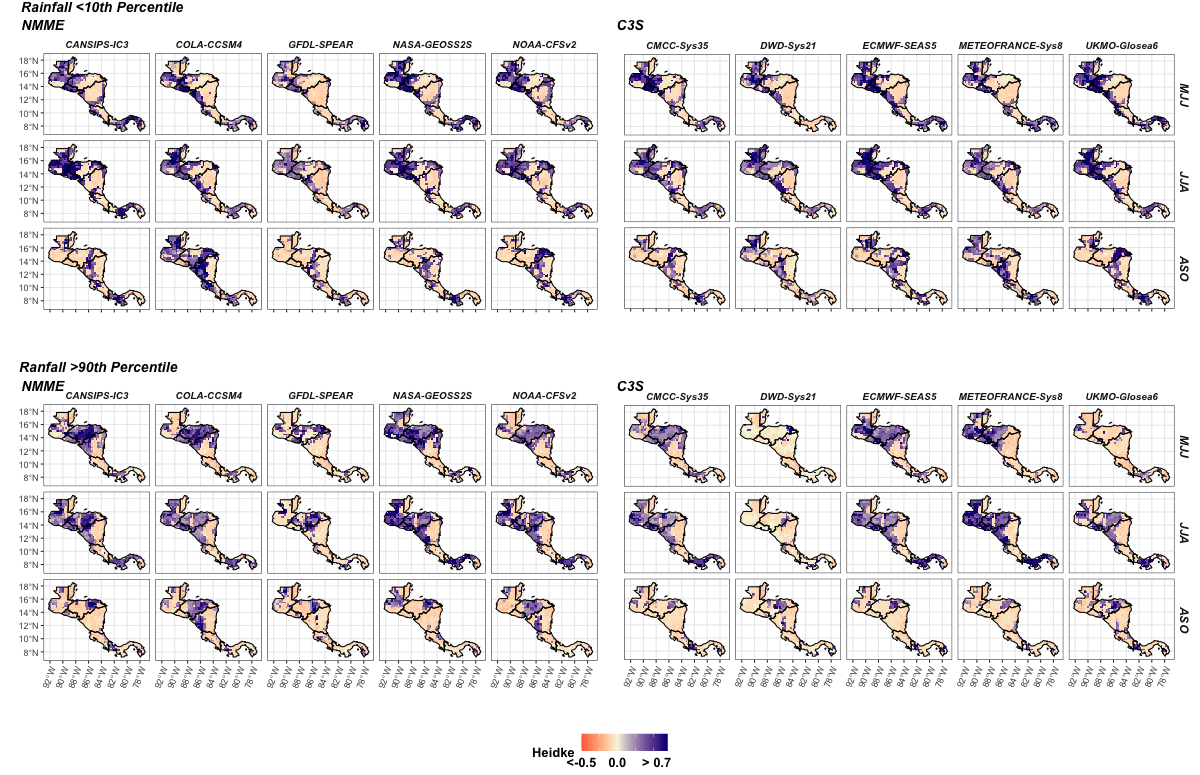


Figure S3 Individual model skill (using Heidke Skill Score) for direct forecasts of low and high rainfall extremes, plotted spatially by wet season period.
